# Are children with IgA nephropathy different from adult patients?

**DOI:** 10.1007/s00467-024-06361-1

**Published:** 2024-04-05

**Authors:** Baige Su, Yuanyuan Jiang, Zhihui Li, Jianhua Zhou, Liping Rong, Shipin Feng, Fazhan Zhong, Shuzhen Sun, Dongfeng Zhang, Zhengkun Xia, Chunyue Feng, Wenyan Huang, Xiaoyan Li, Chaoying Chen, Zhihong Hao, Mo Wang, Li Qin, Minguang Chen, Yuanyuan Li, Juanjuan Ding, Ying Bao, Xiaorong Liu, Fang Deng, Xueqin Cheng, Li Zhang, Xuan Zhang, Huandan Yang, Xiaojie Peng, Qianliang Sun, Linxia Deng, Xiaoyun Jiang, Min Xie, Yan Gao, Lichun Yu, Ling Liu, Chunlin Gao, Jianhua Mao, Weihua Zheng, Xiqiang Dang, Hua Xia, Yujie Wang, Xuhui Zhong, Jie Ding, Jicheng Lv, Hong Zhang

**Affiliations:** 1 Department of Pediatric Nephrology, Peking University First Hospital, No. 1 Xi An Men Da Jie, Beijing, 100034 People’s Republic of China; 2 Renal Division, Peking University First Hospital, No.8 Xi Shi Ku Da Jie, Beijing, 100034 People’s Republic of China; 3 Department of Nephrology, Beijing Hospital of Traditional Chinese Medicine, Capital Medical University, Beijing, China; 4 Department of Nephrology, Rheumatology and Immunology, Hunan Children’s Hospital, Changsha, Hunan China; 5 Department of Pediatrics, Tongji Hospital, Tongji Medical College, Huazhong University of Science & Technology, Wuhan, 430030 Hubei Province China; 6 Department of Pediatric Nephrology and Rheumatology, The First Affiliated Hospital, Sun Yat-Sen University, Guangzhou, China; 7 Department of Pediatric Nephrology, Chengdu Women’s and Children’s Central Hospital, School of Medicine, University of Electronic Science and Technology of China, Chengdu, 611731 China; 8 Pediatric Nephrology Department, Guangzhou Women and Children’s Medical Center, Guangzhou Medical University, Guangzhou, 510623 Guangdong China; 9 Department of Pediatric Nephrology and Rheumatism and Immunology, Shandong Provincial Hospital, Cheeloo College of Medicine, Shandong University, Jinan, 250021 China; 10 Nephrology and Immunology Department, Children’s Hospital of Hebei Province, Shijiazhuang, Hebei Province China; 11 Department of Pediatrics, Jinling Hospital, Medical School of Nanjing University, Nanjing, China; 12 Department of Nephrology, Children Hospital of Zhejiang University School of Medicine, Hangzhou, China; 13 Department of Nephrology and Rheumatology, Shanghai Children’s Hospital, School of Medicine, Shanghai Jiao Tong University, Shanghai, China; 14 Department of Pediatrics, The Second Xiangya Hospital, Central South University, Changsha, China; 15 Department of Nephrology, Children’s Hospital Affiliated to Capital Institute of Pediatrics, Beijing, China; 16 Department of Pediatric, Guangzhou First People’s Hospital, the Second Affiliated Hospital of South China University of Technology, Guangzhou, China; 17 Department of Nephrology, Children’s Hospital of Chongqing Medical University, Chongqing, China; 18 Department of Pediatrics, The First People’s Hospital of Yunnan Province, The Affiliated Hospital of Kunming Science and Technology University, Kunming, China; 19 Department of Pediatric Nephrology, The Second Affiliated Hospital and Yuying Children’s Hospital of Wenzhou Medical University, Wenzhou, China; 20 Department of Pediatrics, Fuzong Clinical Medical College, Fujian Medical University, Fuzhou, 350025 China; 21 Department of Nephrology, Rheumatology and Immunology, Fujian Children’s Hospital, Fuzhou, 350014 China; 22 Department of Pediatric Nephrology, Wuhan Children’s Hospital (Wuhan Maternal and Child Healthcare Hospital), Tongji Medical College, Huazhong University of Science & Technology, Wuhan, 430016 Hubei China; 23 Department of Nephrology, Xi’an Children’s Hospital, Xian, Shaanxi China; 24 Department of Pediatric Nephrology, Beijing Children’s Hospital, Capital Medical University, Beijing, China; 25 Department of Nephrology, Anhui Provincial Children’s Hospital, Hefei, China; 26 Department of Nephrology, Children’s Hospital of Nanjing Medical University, Nanjing, China; 27 Department of Pediatric Nephrology, The First Hospital of Jilin University, Changchun, China; 28 Department of General Medicine, Tianjin Children’s Hospital, Tianjin, China; 29 Department of Nephrology, Xuzhou Children’s Hospital, Xuzhou Medical University, Xuzhou, China; 30 Department of Nephrology, Jiangxi Provincial Children’s Hospital, Nanchang, 330006 China; 31 Medical Data Science Center, Medical Research Center, Beijing Tsinghua Changgung Hospital, School of Clinical Medicine, Tsinghua University, Beijing, China

**Keywords:** IgA nephropathy, Children, Adults, Steroid, Remission of proteinuria

## Abstract

**Background:**

Previously, several studies have indicated that pediatric IgA nephropathy (IgAN) might be different from adult IgAN, and treatment strategies might be also different between pediatric IgAN and adult IgAN.

**Methods:**

We analyzed two prospective cohorts established by pediatric and adult nephrologists, respectively. A comprehensive analysis was performed investigating the difference in clinical and pathological characteristics, treatment, and prognosis between children and adults with IgAN.

**Results:**

A total of 1015 children and 1911 adults with IgAN were eligible for analysis. More frequent gross hematuria (88% vs. 20%, *p* < 0.0001) and higher proteinuria (1.8 vs. 1.3 g/d, *p* < 0.0001) were seen in children compared to adults. In comparison, the estimated glomerular filtration rate (eGFR) was lower in adults (80.4 vs. 163 ml/min/1.73 m^2^, *p* < 0.0001). Hypertension was more prevalent in adult patients. Pathologically, a higher proportion of M1 was revealed (62% vs. 39%, *p* < 0.0001) in children than in adults. S1 (62% vs. 28%, *p* < 0.0001) and T1–2 (34% vs. 8%, *p* < 0.0001) were more frequent in adults. Adjusted by proteinuria, eGFR, and hypertension, children were more likely to be treated with glucocorticoids than adults (87% vs. 45%,* p* < 0.0001). After propensity score matching, in IgAN with proteinuria > 1 g/d, children treated with steroids were 1.87 (95% CI 1.16–3.02, *p* = 0.01) times more likely to reach complete remission of proteinuria compared with adults treated with steroids.

**Conclusions:**

Children present significantly differently from adults with IgAN in clinical and pathological manifestations and disease progression. Steroid response might be better in children.

**Graphical Abstract:**

**Figure:**
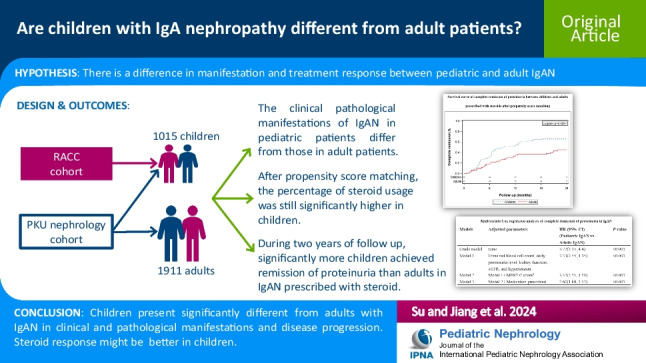
A higher resolution version of the Graphical abstract is available as Supplementary information

**Supplementary Information:**

The online version contains supplementary material available at 10.1007/s00467-024-06361-1.

## Introduction

IgA nephropathy (IgAN) is one of the most common primary glomerulonephritis types worldwide [1, 2], and it has the highest prevalence in Asian people [3–6]. As reported, the incidence of IgAN in Europe was 7.6 cases per million per year and 2.0 cases per million per year in children [7]. But in Japan, the incidence of IgAN was 45 cases per million per year in adults and 99 cases per million per year in children who underwent school urinalysis screening [8–10]. The clinical manifestations of IgAN vary from asymptomatic microscopic hematuria to rapidly progressive glomerulonephritis. Approximately 30% of adult cases with IgAN progressed to chronic kidney disease (CKD) stage 5 in 10 to 20 years [11, 12], while nearly 20% of pediatric IgAN progressed to CKD stage 5 within 20 years of diagnosis [13, 14].

Although both pediatric and adult IgAN are characterized by hematuria, proteinuria, and IgA-predominant deposits pathologically, pediatric and adult nephrologists hold different opinions regarding the treatment of IgAN. Previous retrospective studies from China [15, 16] have shown that glucocorticoids can improve kidney prognosis in children. However, the STOP study on adult IgAN in Europe [17, 18] reported that glucocorticoids and/or immunosuppressants could not delay the progression of kidney function decline. Although the efficacy of glucocorticoids in Asian adults was demonstrated by the TESTING study [19], there has also been increased consideration of their safety. According to the Kidney Disease: Improving Global Outcomes (KDIGO) guideline 2021 [20], it reports as “expert opinion as practice points” the indications for treatment of children that glucocorticoids could be prescribed when the proteinuria > 1 g/d or the protein-to-creatinine ratio (PCR) > 1 g/g. However, for adult patients, a 6-month course of glucocorticoid therapy is suggested only for those at high risk of progressive CKD.

It is important to know whether pediatric and adult IgAN have different clinical–pathological manifestations and treatment responses. However, thus far, there are limited data regarding the difference between pediatric and adult IgAN. Only a retrospective study [21] from China and another small sample study from France [22] have explored the difference in clinical and pathological characteristics between pediatric and adult IgAN. It is unknown whether there is a difference in the treatment response between pediatric and adult IgAN. There is still doubt about the necessity of different treatment strategies for children and adults with IgAN.

Based on the first multicenter prospective cohort of children with IgAN and a large prospective cohort study of adult IgAN patients treated at Peking University First Hospital, we analyzed the differences in clinical and pathological manifestations, treatment patterns and response between pediatric and adult patients with IgAN, aiming to determine whether pediatric patients present differently from adult patients.

## Patients and methods

### Patients

Patients were selected from two prospective cohorts. Since 2016, the Registry of IgA Nephropathy in Chinese Children (RACC) has enrolled children with biopsy-proven IgAN from 28 medical centers in 20 cities.  The cohort from the Department of Nephrology, Peking University First Hospital, includes IgAN patients referred from various centers across China. Informed consent forms were signed by the patients or their guardians. The two cohorts were approved by the ethics committee. The ethics approval numbers are 2013[548] and 2015[992].

Patients were eligible if all the following criteria were met:

1. Patients with a diagnosis of IgAN assessed by kidney biopsy detecting predominant or codominant IgA deposition [20].
2. Patients who were enrolled in the RACC cohort from 2016 to 2021 or in the prospective cohort of IgAN patients established by the Department of Nephrology of Peking University First Hospital from 2003 to 2021.
3. Patients who signed the informed consent form.

Patients who met any of the following criteria were excluded:

1. Patients with systemic diseases, such as IgA vasculitis, systemic lupus erythematosus, and hepatitis B infection.
2. Patients diagnosed with antineutrophil cytoplasmic antibody (ANCA)-associated vasculitis, membranous nephropathy, or tumors.
3. Patients with repeated kidney biopsy whose first pathological diagnosis was not primary IgAN.
4. Patients with an onset age of less than 18 years, and a biopsy age of over 18 years.

Eligible patients were further divided into a pediatric group (< 18 years old) and an adult group (≥ 18 years old).

### Data extraction

Clinical characteristics, pathological diagnoses, prescribed medications, and follow-up data of two years were extracted from the databases of the two cohorts. The pathological Oxford classification [23] was scored by pathologists at each center.

Clinical data were collected at the time of biopsy and during follow-up, including age, sex, weight, height, history of gross hematuria, urine red blood cell count (RBC/µl, urine formed elements analyzer), blood pressure, serum albumin level, serum IgA level, serum creatinine level, and daily urine protein level (proteinuria status was adjusted to g/24 h/1.73 m^2^ for children whose body surface area was less than 1.73 m^2^). The use of medications, including angiotensin-converting enzyme inhibitors, angiotensin receptor blockers, steroids, and immunosuppressive agents (cyclophosphamide, tacrolimus, cyclosporine, leflunomide, and mycophenolate mofetil), was also recorded. The disease duration was defined as the time from disease onset to diagnosis.

The estimated glomerular filtration rate (eGFR) was calculated using the Schwartz equation for children and the Chronic Kidney Disease Epidemiology Collaboration (CKD-EPI) equation for adults. Nephrotic proteinuria was defined as a protein excretion ≥ 3.5 g/d for adults and ≥ 50 mg/kg/d or ≥ 3.5 g/d for children. Nephrotic syndrome was diagnosed in patients with nephrotic proteinuria and a serum albumin level < 3 g/dL. Hypertension was defined as a blood pressure > 140/90 mmHg for adults and an average systolic blood pressure and/or a diastolic blood pressure ≥ 95th percentile (based on age, sex, and height percentiles) [24] for children. Remission of proteinuria was defined as 24-h proteinuria ≤ 200 mg/d [20].

### Statistical analysis

Baseline characteristics are described according to age at the time of biopsy (1015 pediatric IgAN patients; 1911 adult IgAN patients) using frequencies and percentages for categorical variables and medians and interquartile ranges (IQRs) for continuous variables. In the baseline descriptive table, comparisons between pediatric and adult patients were made using chi-squared tests for categorical variables and Mann‒Whitney *U* tests for continuous variables.

Kaplan–Meier estimates and log-rank tests were used to compare the time-to-event clinical outcomes by age and included the following outcomes: (1) time to complete remission and (2) kidney function impairment [25, 26] (time to a 30% reduction in the eGFR or time to a 50% reduction in the eGFR). Furthermore, a multivariate Cox proportional hazards model was used to test the association between the time to complete remission and age, adjusted for baseline 24-h urinary protein concentration, baseline erythrocyte count in hematuria (/µl), baseline eGFR, MEST-C pathological score, hypertension (yes/no), steroid use during follow-up (yes/no), renin–angiotensin–aldosterone system (RAAS) inhibitor use during follow-up (yes/no), and immunosuppression use during follow-up (yes/no).

The two age groups (pediatric vs. adult) showed significant differences in almost all baseline characteristics; therefore, we used greedy nearest neighbor propensity score matching with a 0.25 caliper width to minimize the effects of confounding factors on outcomes when we compared the differences in treatment pattern between pediatric and adult patients whose baseline proteinuria > 1 g/d and the differences in prognosis between pediatric and adult patients whose baseline proteinuria > 1 g/d and all had taken steroid therapy at some point during follow-up. We matched pediatric and adult patients at a 1:1 ratio.

Propensity scores were calculated using a logistic model with the following baseline variables since they showed significant differences according to age and had clinical significance: hypertension (yes/no), eGFR, 24-h urinary protein level, and MEST-C pathological score. To visually compare distributions of balance, a “Standardized Mean Differences” plot was created. The “Standardized Mean Differences” plot shows the differences in the means of matching variables between the children and adults. The differences were standardized by dividing the sample mean by the sample standard deviation, pooled across the child and adult groups. We chose the recommended range of − 0.25 to 0.25 as the good balance limit for the standardized mean differences [27, 28]. Additionally, comparisons and descriptive analyses among the matching variables were conducted for children and adults after propensity score matching to ensure that the matching variables were not significantly different between the children and adults after matching.

A *P* value less than 0.05 was considered to indicate statistical significance. All the statistical analyses were performed using SAS version 9.4 (SAS Institute, Inc., Cary, NC).

## Results

A total of 1015 pediatric patients with IgAN with a median age of 9 years and 1911 adult patients with IgAN with a median age of 32 years were eligible for analysis (see Supplementary Fig. 1).

### Clinical and pathological characteristics of pediatric and adult IgAN

Hematuria and proteinuria were significantly more severe in pediatric IgAN than in adult IgAN. The baseline eGFR was also significantly greater in children than in adults (see Supplementary Fig. 2). Hypertension was more frequent in adults. Pathologically, the proportions of M1 and E1 lesions in children were greater than those in adults. However, S1 and T1–2 lesions were less common in children (Table 1).

**Table 1:** Comparison of clinical and pathological characteristics at biopsy between pediatric and adult IgAN

	Pediatric IgAN	Adult IgAN	*P* value
	(*N* = 1015)	(*N* = 1911)	
Onset age (y)	9 (7, 11)	32 (26, 41)	
Disease duration (m)	1.0 (1.0, 3.0)	16.0 (8.0, 34.0)	< 0.0001
Age at biopsy (y)	10 (7, 12)	34 (28, 43)	
Gender (male, %)	68	50	< 0.0001
History of gross hematuria (%)	88	20	< 0.0001
Hypertension (%)	9	29	< 0.0001
eGFR (ml/min/1.73 m^2^)	163.0 (125.2, 204.1)	80.4 (53.4, 103.6)	< 0.0001
Serum urea (µmol/L)	300.0 (242.8, 381.0)	372.0 (304.0, 445.0)	< 0.0001
ALB (g/L)	33.1 (24.5, 39.4)	38.7 (35.3, 41.7)	< 0.0001
ALB < 30 g/L (%)	40	9	< 0.0001
ALB < 25 g/L (%)	26	5	< 0.0001
Serum IgA (g/L)	2.1 (1.5, 2.9)	3.2 (2.5, 4.0)	< 0.0001
Serum C3 (g/L)	1.1 (0.9, 1.3)	1.0 (0.9, 1.2)	< 0.0001
Urine red blood cell count (/µl)	499.5 (110.0, 2045.0)	12.5 (4.0, 42.5)	< 0.0001
Daily proteinuria (g/24 h/1.73 m^2^)	1.8 (0.8, 3.2)	1.3 (0.7, 2.5)	< 0.0001
Nephrotic proteinuria (%)	55	16	< 0.0001
Nephrotic syndrome (%)	35	6	< 0.0001
MEST-C score*			
M1 (%)	62	39	< 0.0001
E1 (%)	42	34	0.0002
S1 (%)	28	62	< 0.0001
T1-2 (%)	8	34	< 0.0001
C1-2 (%)	52	59	0.0017

*Pathological Oxford classification was available for 788 children and 1389 adults

*IgAN* IgA nephropathy, *eGFR* estimated glomerular filtration rate, *ALB* albumin

### Comparison of medication use between pediatric and adult IgAN

The proportion of patients prescribed corticosteroids alone or in combination with other immunosuppressants was significantly greater in children with IgAN than in adults with IgAN (Table 2).

**Table 2:** Comparison of treatment pattern between children and adults

	Pediatric IgAN	Adult IgAN	*P* value
	(*N* = 1015)	(*N* = 1911)	
CSs (%)	74	40	< 0.0001
Only CSs (%)	21	14	< 0.0001
CSs + CTX (%)	13	7	< 0.0001
CSs + MMF (%)	10	1	< 0.0001
CSs + TAC (%)	6	0	< 0.0001
CSs + etc.* (%)	7	7	0.6295
RAS blockers (%)	49	94	< 0.0001

*IgAN* IgA nephropathy, *CSs* corticosteroids, *CTX* cyclophosphamide, *MMF* mycophenolate mofetil, *TAC* tacrolimus, *RAS* renin-angiotensin system

*Etc., cyclosporine, leflunomide, or triptolide

### Disease prognosis of pediatric and adult IgAN

Overall, 268 pediatric patients and 1557 adult patients with IgAN were followed up for more than 2 years.

#### Complete remission of proteinuria

**Fig. 1:**
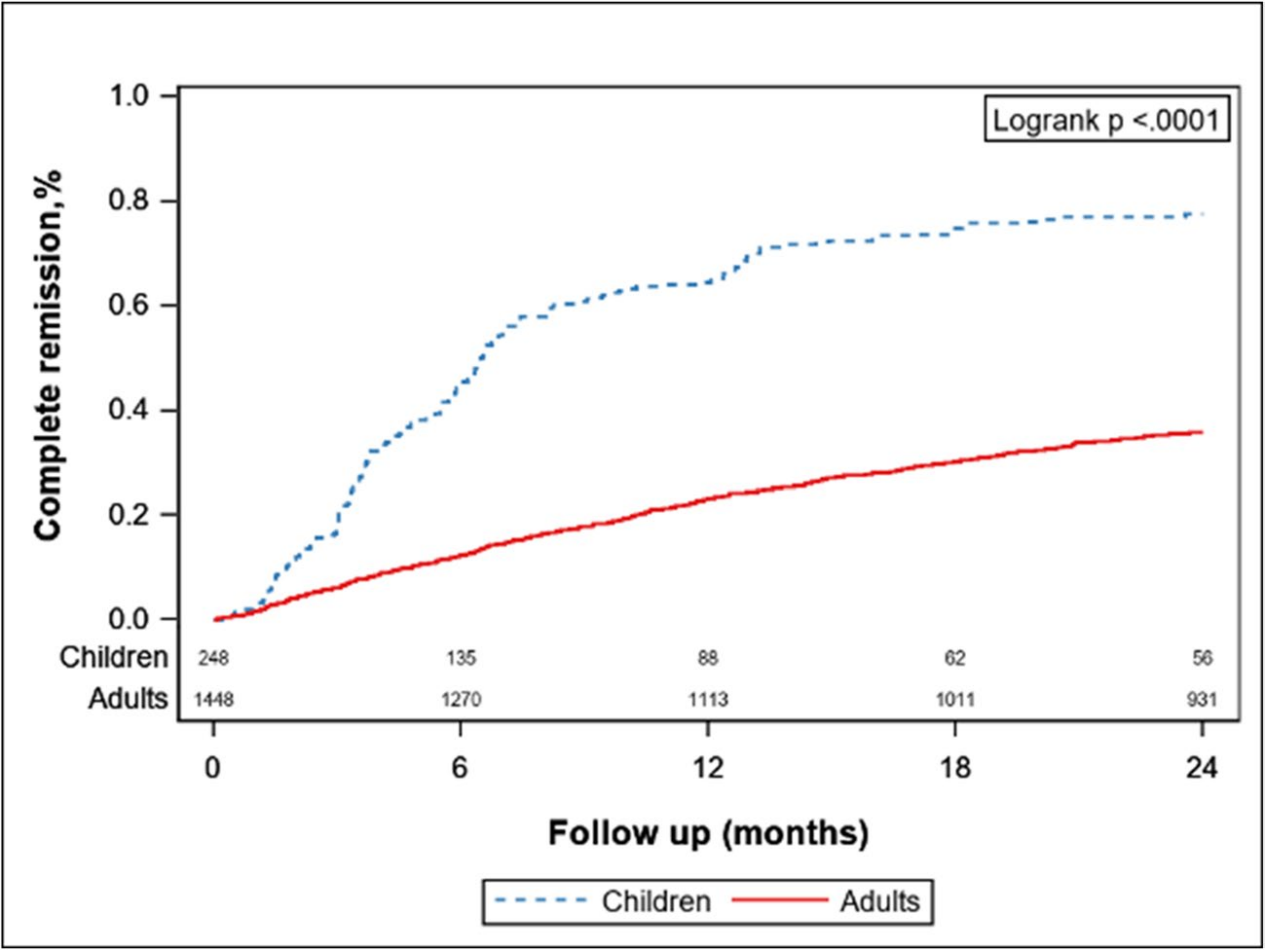
Comparison of the complete remission rate of proteinuria between children and adults

The median follow-up durations of the children and adults were 29 (24, 45.5) months and 64 (40, 102.2) months, respectively. During 2 years of follow-up, 192 (77.42%) children and 517 (35.7%) adults with IgAN experienced complete remission of proteinuria (*p* < 0.001; Fig. 1). The median time from biopsy to complete remission was 5.18 months and 8.88 months in children and adults, respectively.

After multivariate analysis, the probability of complete remission of proteinuria in pediatric IgAN was still significantly greater than that in adult IgAN (HR, 2.6; 95%CI, 1.89–3.57; *p* < 0.001) (Table 3).

**Table 3:** Multivariate Cox regression analysis of complete remission of proteinuria in IgAN

Models	Adjusted parameters	HR (95% CI) (pediatric IgAN vs. adults IgAN)	*P* value
Crude model	None	3.72 (3.15, 4.4)	< 0.001
Model 1	Urine red blood cell count, daily proteinuria level, kidney function, eGFR, and hypertension	3.33 (2.55, 4.35)	< 0.001
Model 2	Model 1 + MEST-C score^#^	3.37 (2.51, 4.51)	< 0.001
Model 3	Model 2 + Medication prescribed	2.60 (1.89, 3.57)	< 0.001

Medication prescribed contained three variables, steroid use (yes/no), renin–angiotensin–aldosterone system (RAAS) inhibitor use (yes/no), and immunosuppressant use (yes/no)

^#^MEST-C score, M1, E1, S1, T1-2, C1-2

#### eGFR decline

During two years of follow-up, a total of 48 (19.05%) pediatric patients with IgAN and 465 (32.91%) adult patients with IgAN experienced a 30% decrease in the eGFR. Among these patients, the eGFR of pediatric patients with IgAN reached a 30% decrease more slowly than did that of adult patients (*p* < 0.01; Supplementary Fig. 3a). There was no significant difference in the decrease in the eGFR by 50% between children with IgAN and adults with IgAN (*p* = 0.14; Supplementary Fig. 3b).

### Steroid response in pediatric and adult IgAN

#### Medication prescribed in the subgroup of patients with proteinuria > 1 g/d after propensity score matching

Among the patients followed up for more than 2 years, 193 children and 892 adults presented with proteinuria > 1 g/d at baseline. Children were more likely to be treated with glucocorticoids than adults were (87% vs. 45%) (Table 4).

**Table 4:** Baseline characteristics and medications prescribed for pediatric and adult IgAN after propensity score matching

	Pediatric IgAN (*N* = 93)	Adult IgAN (*N* = 93)	*P* value
Hypertension (%)	13 (14)	18 (19)	0.33
eGFR (ml/min/1.73 m^2^)	119.9 (94.0, 136.0)	116.3 (104.8, 125.7)	0.16
Proteinuria (g/24 h/1.73 m^2^)	2.2 (1.5, 3.8)	2.1 (1.4, 2.9)	0.52
M1, *N* (%)	45 (48)	44 (47)	0.88
E1, *N* (%)	41 (44)	39 (42)	0.77
S1, *N* (%)	42 (45)	47 (51)	0.46
T1-2, *N* (%)	15(16)	15(16)	1.00
C1-2, *N* (%)	60(65)	62(67)	0.29
Medication prescribed			
CSs (%)	81 (87)	42 (45)	< .0001
Only CSs (%)	31 (33)	18 (19)	0.03
CSs + CTX (%)	9 (10)	9 (10)	1.00
CSs + MMF (%)	6 (6)	0 (0)	0.03
CSs + TAC (%)	6 (6)	1 (1)	0.12
CSs + etc.^#^ (%)	13 (14)	4 (4)	0.02
RAS blockers (%)	67 (72)	87 (94)	< .001

*CSs* corticosteroids, *CTX* cyclophosphamide, *MMF* mycophenolate mofetil, *TAC* tacrolimus

^#^Etc., cyclosporine, leflunomide, or triptolide

**Fig. 2:**
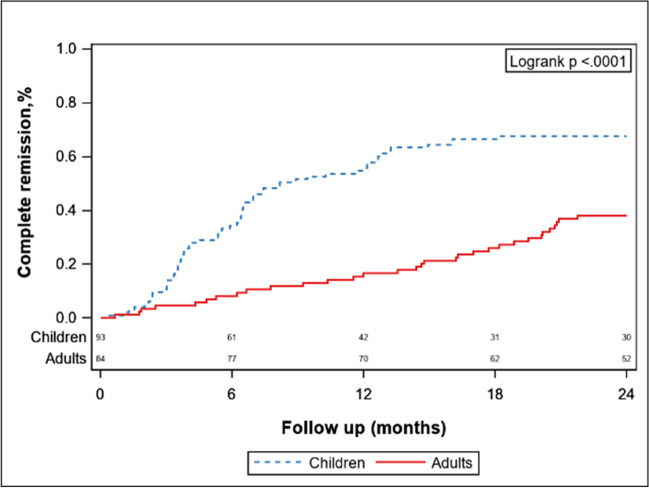
Survival curve of patients with complete remission of proteinuria between the pediatric and adult groups with proteinuria > 1 g/d after propensity score matching

The percentage of patients in complete remission of proteinuria was significantly greater in the pediatric group than in the adult group (67.74% vs. 38.1%) (HR, 2.78; 95% CI, 1.81–4.26, *p* < 0.001) (see Fig. 2). Children were less likely to experience a 30% decrease in the eGFR from baseline than adults were (13.48% vs. 23.66%, *p* < 0.01) (see Supplementary Fig. 4a). Among these patients, the eGFR of children with IgAN reached a 30% decrease more slowly than did that of adults with IgAN. There was no significant difference in the decrease in the eGFR by 50% between children with IgAN and adults with IgAN (Supplementary Fig. 4b, *p* = 0.29).

#### Steroid response in the subgroup of patients with proteinuria > 1 g/d after propensity score matching

Among the 193 children and 892 adults with proteinuria > 1 g/d, 167 children and 464 adults were prescribed steroids, respectively. The results were matched by propensity score (Table 5).

**Table 5:** Baseline characteristics of pediatric and adult IgAN prescribed steroids after propensity score matching

	Pediatric IgAN (*N* = 68)	Adult IgAN (*N* = 68)	*P* value
Hypertension (%)	9 (13)	16 (24)	0.12
eGFR (ml/min/1.73 m^2^)	114.0 (86.0, 130.9)	106.9 (78.7, 119.4)	0.06
Proteinuria (g/24 h/1.73 m^2^)	2.5 (1.5, 4.7)	2.0 (1.3, 4.3)	0.25
M1, *N* (%)	32 (47)	33 (49)	0.86
E1, *N* (%)	30 (44)	29 (43)	0.86
S1, *N* (%)	31 (46)	32 (47)	0.86
T1-2, *N* (%)	11 (16)	15 (22)	0.66
C1-2, *N* (%)	42 (62)	43 (64)	0.85
Steroid^#^	68 (100)	68 (100)	-

^#^Patients treated with steroids with or without other immunosuppressive agents

**Fig. 3:**
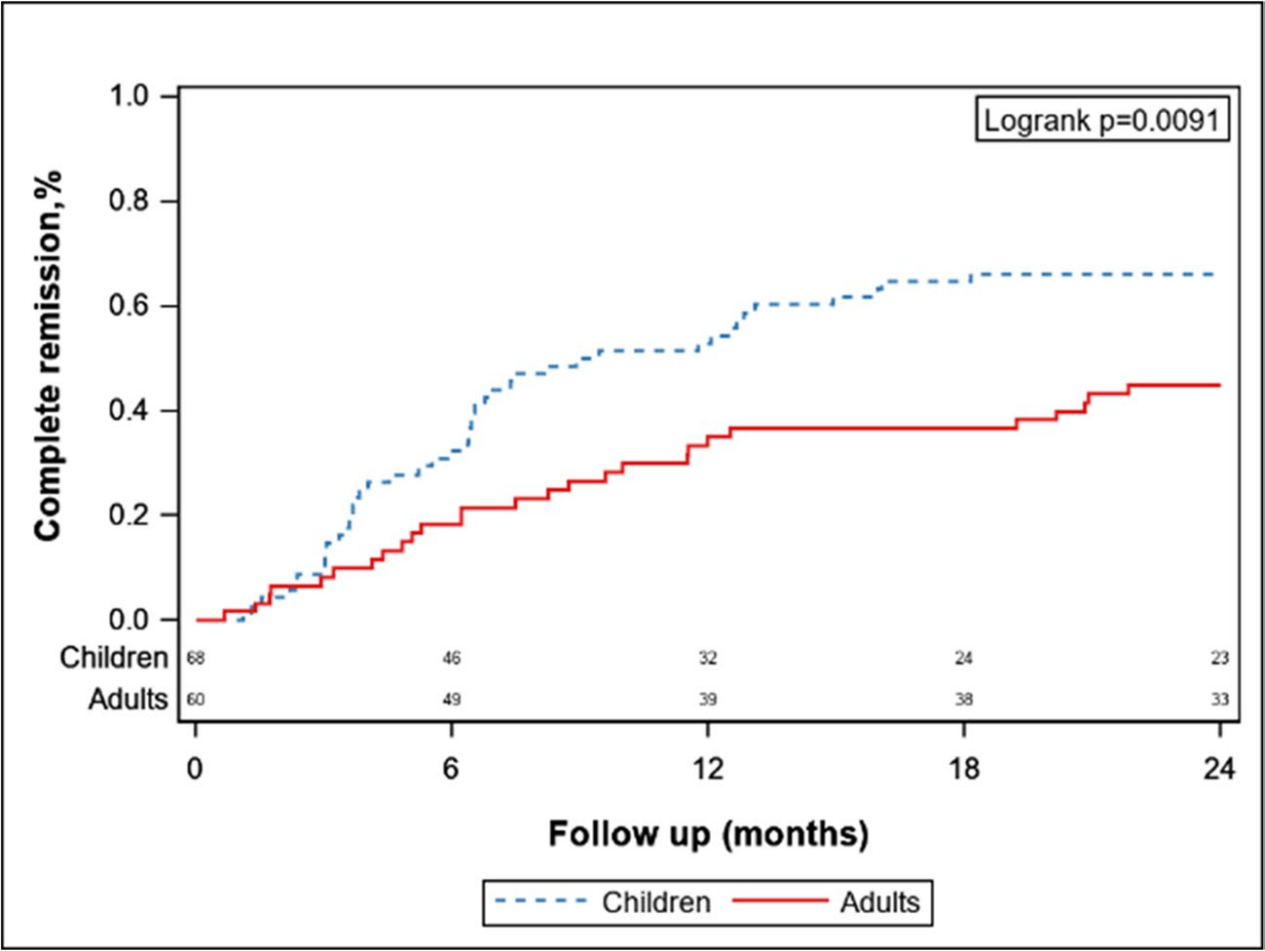
Survival curve of complete remission of proteinuria between children and adults prescribed with steroids after propensity score matching

Children treated with steroids were more likely to reach complete remission of proteinuria in two years than adults were (HR, 1.87; 95% CI, 1.16–3.02; *p* = 0.01) (see Fig. 3). During the follow-up, the eGFR of children with IgAN reached a 30% decrease more slowly than did that of adults with IgAN (*p* < 0.01; Supplementary Fig. 5a). There was no significant difference in the decrease in the eGFR by 50% between children with IgAN and adults with IgAN (*p* = 0.13; Supplementary Fig. 5b).

## Discussion

This study included two prospective cohorts established by pediatric and adult nephrologists. A total of 2926 patients with IgAN, including 1015 children and 1911 adults, were included. The RACC cohort was established in 2016 as the first multicenter prospective cohort study of pediatric IgAN patients in China. The adult cohort was established in 2003. This is one of the largest multicenter studies of IgAN patients in China in which patients were referred from various centers across China. A comprehensive analysis was performed to investigate the differences in clinical and pathological characteristics, treatment, and prognosis between children and adults with IgAN.

In our study, nearly 90% of the children with IgAN had a history of gross hematuria, which was significantly greater than the percentage of adults. Gross hematuria is the most common symptom of IgAN in children reported in different countries [14, 22, 29–31] and may be related to prodromal infection [21]. We revealed that the prevalence of nephrotic proteinuria was 55% in children, which was higher than 20% in the VALIGA study [32], and 27% in the Oxford study [31]. This may be related to differences in geography, race, and kidney biopsy indications. While in a Japanese study [33], the percentage of nephrotic syndrome in pediatric patients with IgAN (7%) was much lower than our study and other areas. This may be related to the national screening school program in Japan.

Based on previous limited data, hematuria is more common in children. However, the difference in the incidence of proteinuria is unclear. In our study, compared with adult IgAN, pediatric IgAN presented with more severe proteinuria, greater daily proteinuria levels and a greater incidence of nephrotic syndrome. In addition, impaired kidney dysfunction and hypertension were more common in adults with IgAN. Pathologically, pediatric IgAN is more commonly characterized by active lesions, such as mesangial proliferation and endocapillary hypercellularity. Chronic diseases such as segmental glomerulosclerosis, tubular atrophy, and interstitial fibrosis are more common in adult patients with IgAN. Therefore, the clinical pathological manifestations of IgAN in pediatric patients differ from those in adult patients.

To date, there are few data on the differences in treatment patterns and treatment responses between children and adults. In our study, more than half of the children were treated with RAAS blockers routinely, while almost all adult patients were treated with RAAS blockers. RAAS blockers were reported to be widely used in children both in Japan (95%) and Europe (86%) [32, 34]. Our study showed that more than 80% of pediatric patients were treated with glucocorticoids and/or immunosuppressants in clinical practice, while only approximately 40% of adult patients were treated with steroids. After adjustment for proteinuria, eGFR, and hypertension, the prevalence of steroid use was still significantly greater in children. Thus, pediatric and adult nephrologists tend to give different prescriptions even if the patients have comparable proteinuria, kidney function, and pathological classifications. Nevertheless, why a higher percentage of children were treated with steroids is not clear to us and needs future study.

Previous studies on the treatment of IgAN have drawn different conclusions for adults and children considering the indications for steroids. A retrospective pediatric report from China [16] supported the use of steroids in pediatric IgAN patients with proteinuria ≥ 1 g/d. The STOP study [17, 18] demonstrated that immunosuppressive therapy could not improve the outcomes of adult patients with proteinuria > 0.75 g/d. However, the TESTING study [19] revealed that oral steroids could prevent the risk of the composite outcome of kidney function impairment or death in adult patients with proteinuria > 1 g/d. The KDIGO 2021 guidelines gave different treatment recommendations for children and adults with IgAN [20]. It mentioned that children with IgAN could be prescribed glucocorticoids when the proteinuria > 1 g/d or the PCR > 1 g/g. However, for adult patients, a 6-month course of glucocorticoid therapy is suggested only for those at high risk of progressive CKD despite maximal supportive care. However, there is still a lack of evidence for the use of different treatment strategies for pediatric and adult IgAN patients.

Furthermore, our study investigated the steroid response in IgAN patients with proteinuria > 1 g/d in both age groups. A comparison of the difference in therapeutic efficacy in patients with proteinuria > 1 g/d was performed after propensity score matching. Children treated with steroids were 1.87 times more likely to reach complete remission of proteinuria than adults treated with steroids. The median time from diagnosis to complete remission was also significantly shorter in pediatric IgAN than in adult IgAN. Data on the time elapsed from gross hematuria to kidney biopsy was not collected. This might influence the detection of active lesions as well as the high rate of remission. Therefore, by multivariate analysis, in IgAN patients treated with steroids, children are significantly more likely to reach remission of proteinuria within 2 years of follow-up compared with adults. This finding supports the implementation of different treatment strategies in children and adults with IgAN.

Our study has several limitations. First, as an observational study, bias, confounders, and missing data were inevitable. The patients were enrolled in the two cohorts at different time points. The indications for kidney biopsy and medications prescribed might vary among centers. The number of pediatric patients included in the survival analyses was limited. To reduce the potential effects of bias, propensity score matching was performed. Further interventional studies are necessary to confirm or compare the efficacy of the steroids in two populations. Only 2-year follow-up data was used to compare the two age groups. Long-time follow-up study is still needed in the future. Second, safety data were not collected. Only treatment efficacy data were analyzed. Third, the response to immunosuppressive agents was not studied. The appropriate dose and course of treatment were also not evaluated. All the above questions need to be further explored in future studies.

In summary, by analyzing the two prospective cohorts established by pediatric and adult nephrologists, this study demonstrated significant differences in the clinicopathological phenotypes between children and adults with IgAN. Pediatric IgAN patients are more likely to present with severe hematuria, nephrotic proteinuria, or proliferative lesions than adult IgAN patients are. However, adult patients are more likely to present with kidney dysfunction, hypertension, and chronic lesions than pediatric patients are. Furthermore, we identified differences in the treatment pattern and steroid response between pediatric and adult IgAN. Therefore, children with IgAN present differently from adult patients. Different treatment strategies should be implemented for children and adults. This is very important when we face emerging therapies for IgAN, for which we need to make a decision.

### Supplementary Information

Below is the link to the electronic supplementary material.

Graphical abstract (PPTX 191 KB)

Supplementary file2 (DOCX 628 KB)

## Data Availability

For protecting study participant privacy, our data cannot be shared openly.
