# Supplementary material for: Are children with IgA nephropathy different from adult patients?
Source: Pediatr Nephrol. 2024 Apr 5;39(8):2403–12. doi: 10.1007/s00467-024-06361-1 (PMC11199250; doi:10.1007/s00467-024-06361-1)
Supplement: Supplementary file 1 — Graphical abstract (PPTX 191 KB) [file 467_2024_6361_MOESM1_ESM.pptx]

## Slide 1
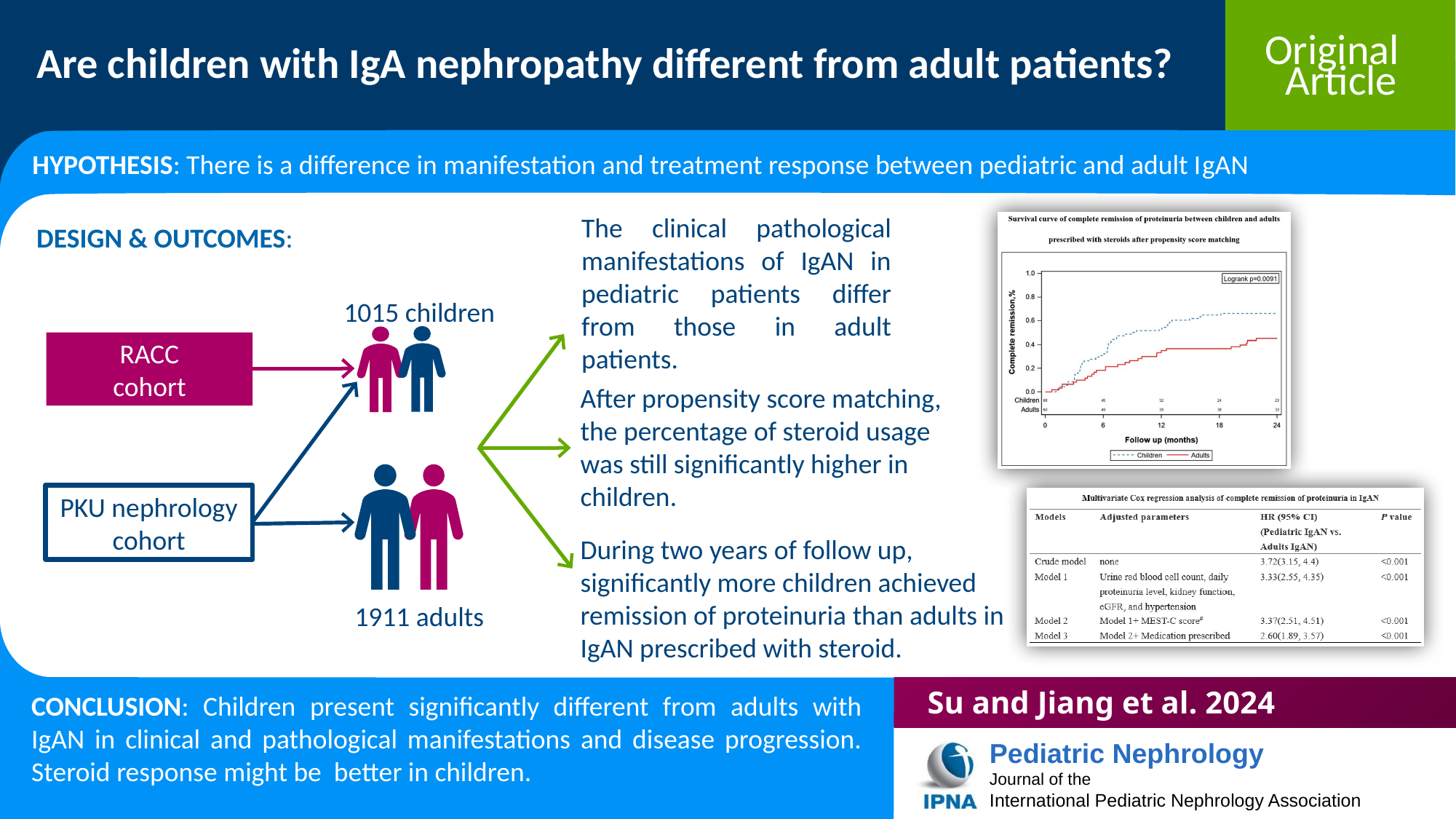

Are children with IgA nephropathy different from adult patients?
HYPOTHESIS: There is a difference in manifestation and treatment response between pediatric and adult IgAN
DESIGN & OUTCOMES:
The clinical pathological manifestations of IgAN in pediatric patients differ from those in adult patients.
1015 children
RACC
cohort
After propensity score matching,
the percentage of steroid usage was still significantly higher in children.
PKU nephrology cohort
During two years of follow up, significantly more children achieved remission of proteinuria than adults in IgAN prescribed with steroid.
1911 adults
Su and Jiang et al. 2024
CONCLUSION: Children present significantly different from adults with IgAN in clinical and pathological manifestations and disease progression. Steroid response might be better in children.
