# Supplementary material for: Are children with IgA nephropathy different from adult patients?
Source: Pediatr Nephrol. 2024 Apr 5;39(8):2403–12. doi: 10.1007/s00467-024-06361-1 (PMC11199250; doi:10.1007/s00467-024-06361-1)
Supplement: Supplementary file 2 — Supplementary file2 (DOCX 628 KB) [file 467_2024_6361_MOESM2_ESM.docx]

**Supplementary material**

**Supplementary Figure 1** Flow chart of the inclusion and exclusion of patients

**Supplementary Figure 2** Histograms for eGFR at baseline of two age groups

**Supplementary Figure 3** (a) A 30% eGFR decline or more in pediatric and adult IgAN within 2 years (*p* < 0.01)

(b) A 50% eGFR decline or more in pediatric and adult IgAN within 2 years (*p* = 0.14)

**Supplementary Figure 4** (a) A 30% eGFR decline or more in pediatric and adult IgAN with proteinuria >1 g/d after propensity score matching (*p*<0.01)

(b) A 50% eGFR decline or more in pediatric and adult IgAN with proteinuria >1 g/d after propensity score matching (*p*=0.29)

**Supplementary Figure 5** (a)A 30% eGFR decline or more in pediatric and adult IgAN prescribed steroids after propensity score matching (*p*<0.01)

(b) A 50% eGFR decline or more in pediatric and adult IgAN prescribed steroids after propensity score matching (*p*=0.13)

**Supplementary Figure 6** (a) A 30% eGFR decline or more in pediatric and adult IgAN entire follow up (*p* < 0.01)

(b) A 50% eGFR decline or more in pediatric and adult IgAN entire follow up (*p* = 0.07)

**Supplementary table 1** Multivariate Cox regression analysis of complete remission of proteinuria in IgAN

**Supplementary Figure 1**


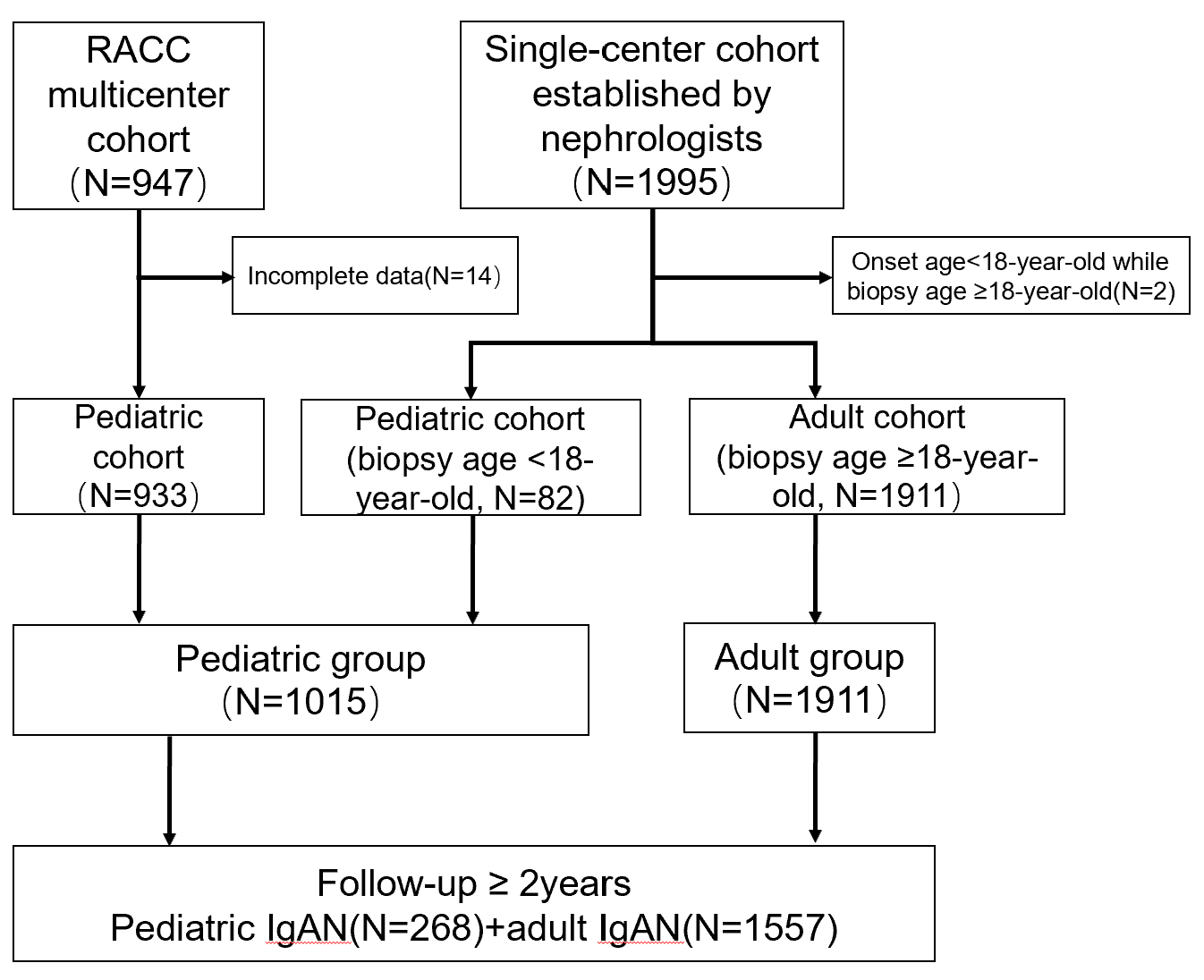


**Supplementary Figure 2**

**
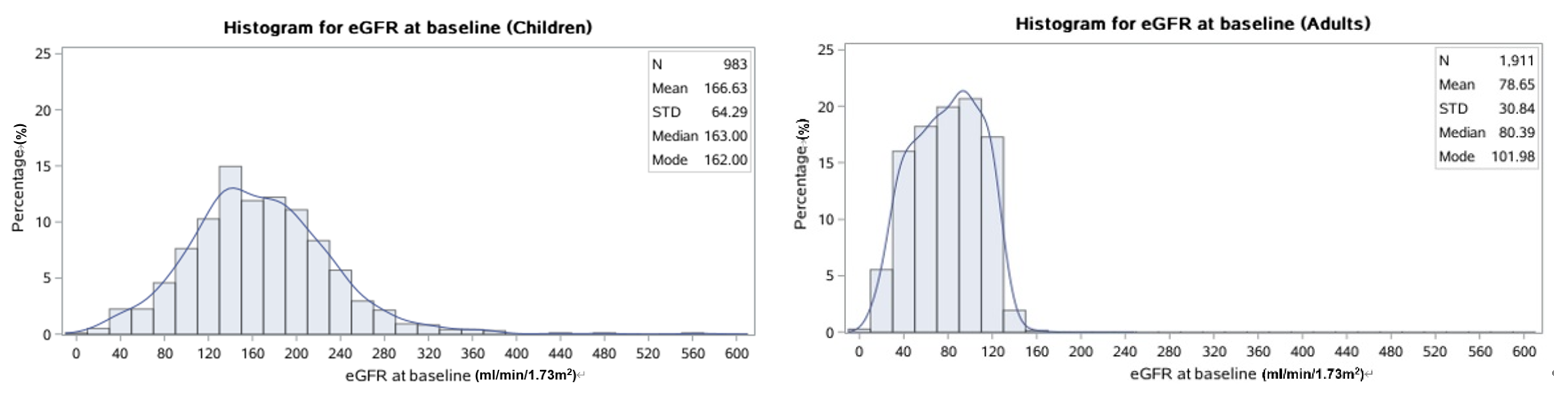
**

**Supplementary Figure 3 (a)(b)**

**
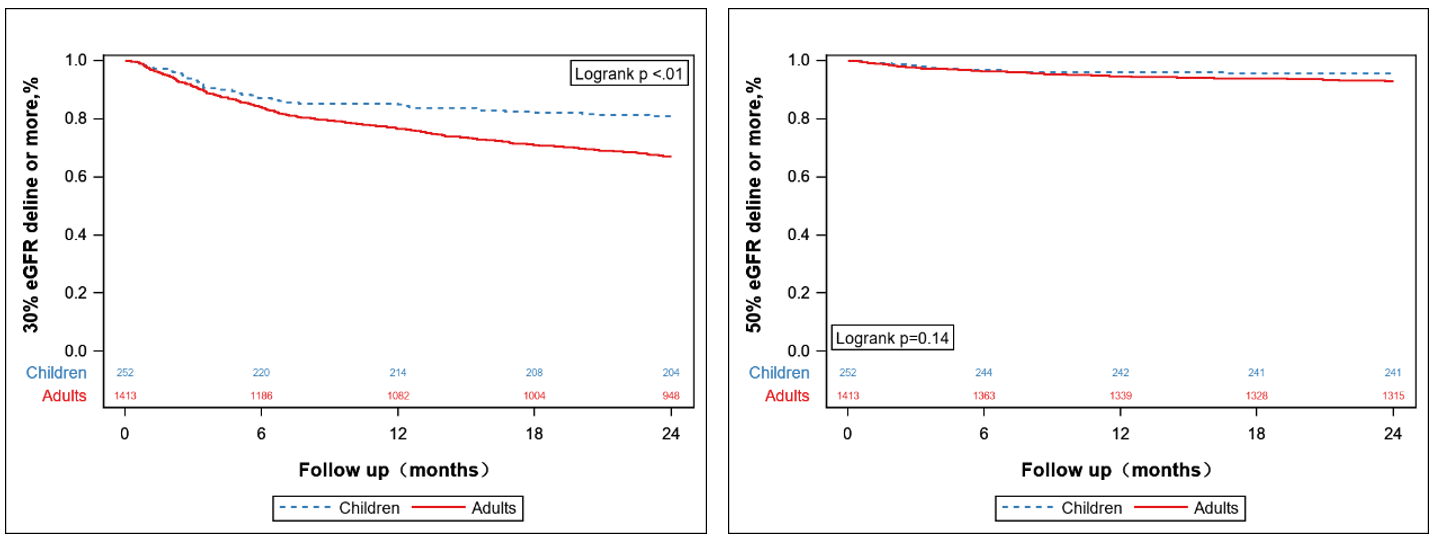
**

**Supplementary Figure 4 (a)(b)**

**
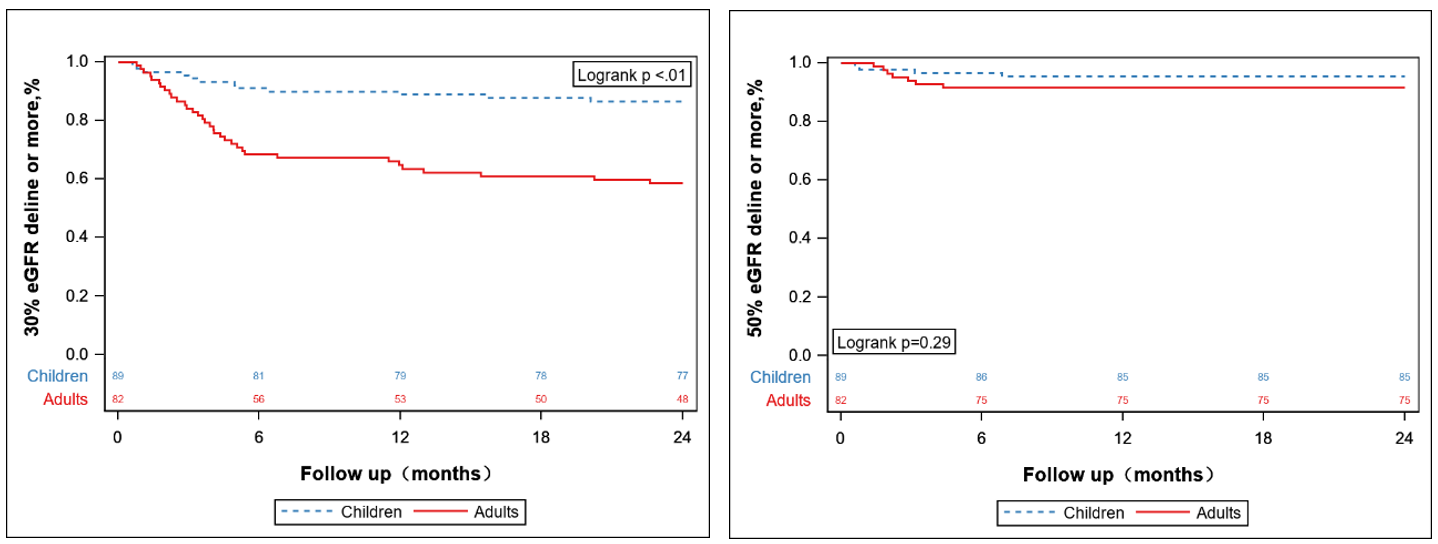
**

**Supplementary Figure 5 (a)(b)**

**
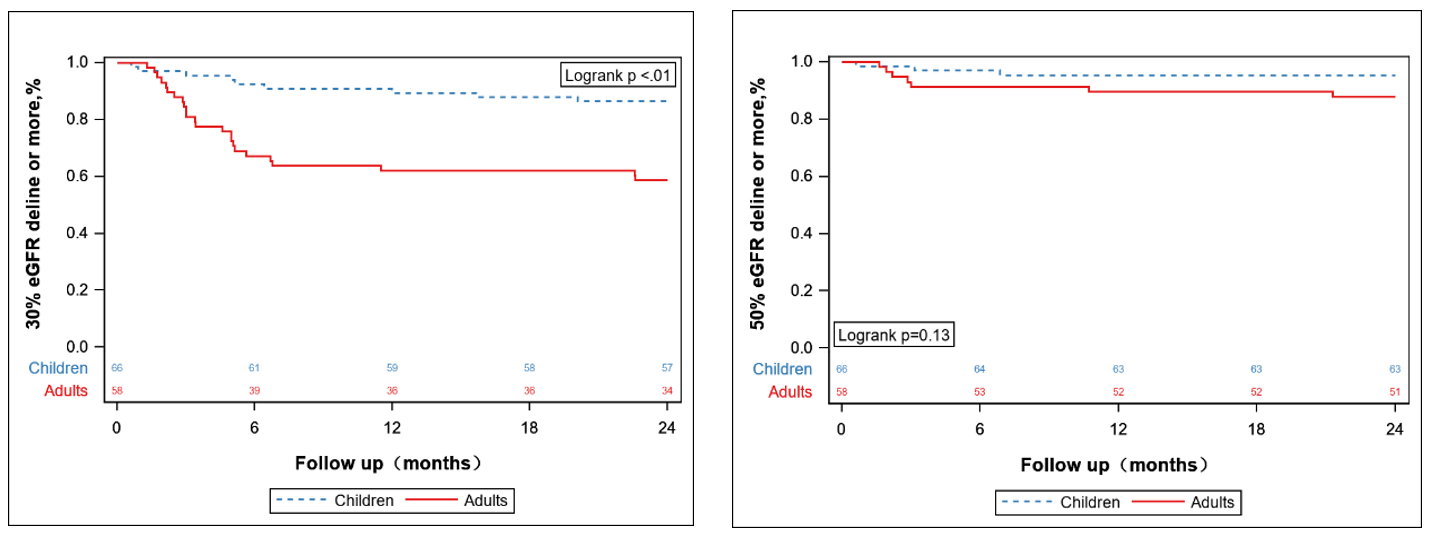
**

**Supplementary Figure 6 (a)(b)**


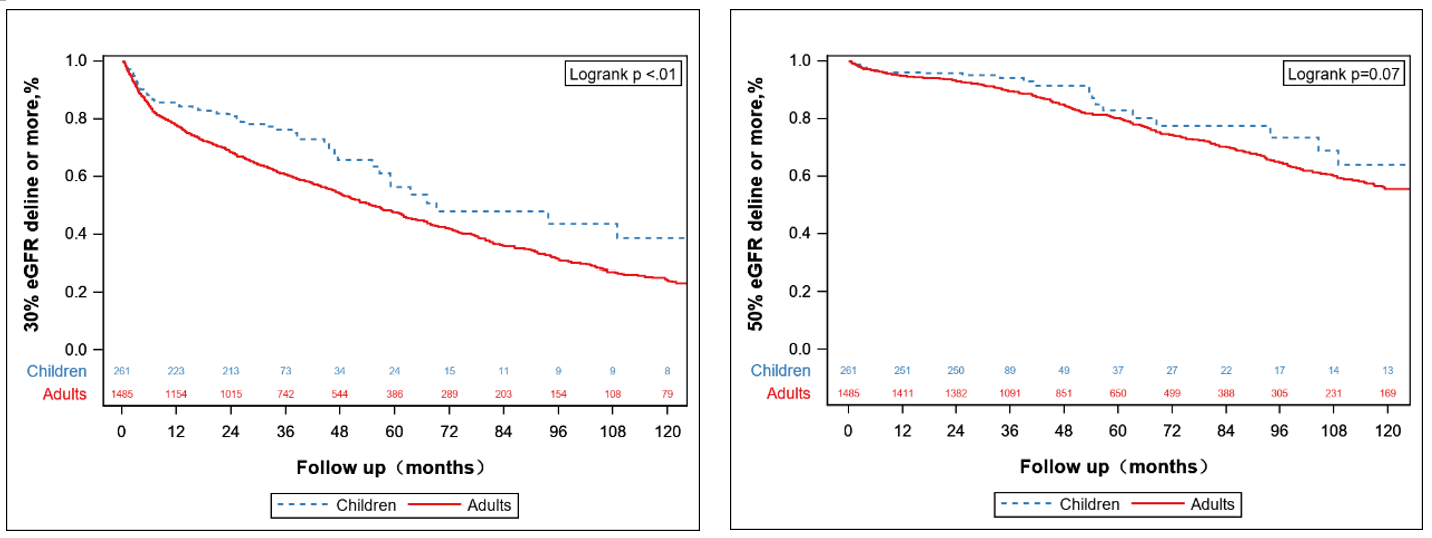


**Supplementary table 1**

|  | **HR** | **95% CI** | ***P*** |
| --- | --- | --- | --- |
| **Children vs. Adults** | 2.60 | 1.89 to 3.57 | <0.001 |
| baseline 24-hour urinary protein concentration | 0.91 | 0.86 to 0.97 | 0.003 |
| baseline erythrocyte count in hematuria (/μl)  (per 100/μl increase) | 1.00 | 1.00 to 1.01 | 0.21 |
| baseline eGFR (per 10ml/min/1.73m^2^ increase) | 1.01 | 0.98 to 1.03 | 0.64 |
| hypertension (yes/no) | 1.11 | 0.88 to 1.39 | 0.39 |
| M1: 0 vs. 1 | 1.00 | 0.81 to 1.22 | 0.97 |
| E1: 0 vs. 1 | 0.89 | 0.72 to 1.08 | 0.24 |
| S1: 0 vs. 1 | 1.39 | 1.14 to 1.71 | 0.001 |
| T1: 0 vs. 2 | 1.20 | 0.79 to 1.82 | 0.38 |
| T1: 1 vs. 2 | 1.03 | 0.68 to 1.57 | 0.88 |
| C1: 0 vs. 2 | 1.17 | 0.84 to 1.62 | 0.36 |
| C1: 1 vs. 2 | 1.04 | 0.76 to 1.42 | 0.82 |
| Steroid use during follow-up (yes/no) | 2.86 | 1.87 to 4.36 | <0.001 |
| Renin-angiotensin-aldosterone system (RAAS) inhibitor use during follow-up (yes/no) | 0.75 | 0.54 to 1.04 | 0.09 |
| Immunosuppression use during follow-up (yes/no) | 0.51 | 0.33 to 0.76 | 0.001 |
